# From Osteogenesis to Diagnosis: The Role of microRNAs as Biomarkers for Osteoporosis

**DOI:** 10.3390/ijms27031158

**Published:** 2026-01-23

**Authors:** Qinyong You, Yifan Niu, Zhiyu Lu, Ziyuan Wang, Runting Li, Jiaming Zhang, Yun Tian, Tengjiao Zhu

**Affiliations:** 1 Department of Orthopedics, Peking University Third Hospital, Beijing 100191, China; 2511210385@stu.pku.edu.cn (Q.Y.); xiaoniuyf0601@163.com (Y.N.); 2010301330@stu.pku.edu.cn (Z.L.); 1810301340@pku.edu.cn (Z.W.); lrt@pku.edu.cn (R.L.); 2 Engineering Research Center of Bone and Joint Precision Medicine, Peking University Third Hospital, Beijing 100191, China; 3 Clinical Medicine, Medical College, Yanbian University, Yanji 133002, China; zhangjiamingybdx@126.com

**Keywords:** microRNAs, osteoporosis, osteoblast, diagnostic biomarkers

## Abstract

With the progress of global aging, osteoporosis, as a systemic bone disease, has become an increasingly serious public health problem. Osteoporosis has an insidious onset, and the fractures it causes have a high rate of disability and mortality. Early diagnosis and intervention of the disease are particularly important. Currently, diagnostic methods for osteoporosis, such as dual-energy X-ray absorptiometry (DXA), quantitative computed tomography (QCT), and bone turnover markers (BTM), all have their limitations. miRNA is a type of non-coding RNA that plays a role in the epigenetic regulation of gene expression. A large number of studies have shown that miRNA is involved in the formation and functional execution of osteoblasts. The differential expression of miRNA levels can effectively distinguish osteoporosis patients from normal individuals, and miRNA detection has the advantages of simple sample collection, non-invasive measurement, specificity for bone metabolism, correct correlation with standard techniques for bone remodeling analysis, and the ability to respond to the treatment of diseases affecting bone metabolism. This makes miRNAs potentially effective diagnostic markers for osteoporosis. This article aims to summarize our current understanding of miRNA regulation of osteoblast generation and function, and we will also discuss the potential value of these miRNAs as biomarkers for the diagnosis of osteoporosis.

## 1. Introduction

Osteoporosis is defined as a systemic bone disease characterized by the loss of bone mass and destruction of bone microstructure, resulting in increased bone fragility and vulnerability to fractures [1]. The global prevalence of osteoporosis and bone loss was 19.7% and 40.4%, respectively. The prevalence in developing countries (22.1%) was higher than that in developed countries (14.5%) [2]. Low bone density (LBMD), including osteoporosis and osteopenia (low bone mass), affects nearly 200 million people worldwide, and the number of deaths related to LBMD is continuously increasing globally [3]. The onset of osteoporosis is insidious. Early identification of patients with osteoporosis is the key to effective treatment. The quantitative analysis of bone mineral density (BMD) by dual-energy X-ray absorptiometry (DXA) has been widely used as the gold standard for the diagnosis of osteoporosis. The WHO defines osteoporosis as a BMD lower than 2.5 SD T value of the mean (<−2.5 SD) [4]. However, the utility of BMD as a clinical indicator of osteoporosis is limited; BMD is only one of a number of important risk factors for fractures, and most fragility fractures occur in individuals with BMD values above this threshold [1]. In addition, DXA can only provide local bone strength information, while osteoporosis is a systemic bone disease characterized by bone fragility [5]. What is more, osteophytes, ligament calcification, spinal degeneration, or abdominal aortic calcification in the lumbar spine region can lead to an overestimation of BMD by DXA. For example, quantitative computed tomography (QCT) has also been used to determine bone mineral density (BMD) to evaluate osteoporosis. Compared with DXA, QCT has higher sensitivity for the detection of OP [6]. However, this imaging method still has its limitations, such as ionizing radiation exposure, large machine size, high cost, and low accessibility. It is still necessary to explore new methods for early identification of osteoporosis [5]. Furthermore, bone turnover markers (BTMs) have been widely studied and used to evaluate the rate of bone formation and resorption. Commonly used bone formation markers (bone alkaline phosphatase, osteocalcin, and N-terminal propeptide type I procollagen) and commonly used resorption markers (serum C-telopeptide of type I collagen, N-telopeptide of type I collagen, and tartrate-resistant acid phosphatase isoform 5b) can effectively reflect bone metabolism. In addition, BTM can be measured in blood and urine, which has the advantages of convenience and non-invasiveness. Therefore, BTM can be used as supplementary information for the diagnosis of osteoporosis. However, BTM levels are affected by factors such as age, sex, race, fasting/feeding status, and time relative to circadian rhythm, menstrual cycle, exercise, pregnancy, season, medications, and recent fractures [7]. In conclusion, it is necessary to explore new sensitive and specific biomarkers for early identification of osteoporosis and assessment of fracture risk.

MiRNAs are small single-stranded RNAs, 18~25 nucleotides in length. miRNA is not translated into protein. Its function is to bind to the 3′-untranslated region (3′ -UTR) of the target mRNA and inhibit the expression of the target protein gene [6]. MiRNAs have been widely studied as important intervention targets and prediction tools for various diseases due to their high conservation and detection stability [8], for example, colorectal cancer, bladder cancer, tuberculosis, intracranial aneurysms, etc. [9,10,11,12]. MiRNA, as an important intracellular signaling molecule, plays an epigenetic regulatory role in gene expression. It is known that miRNA is involved in various biological processes, including bone resorption and bone formation. Studies have shown that the differential expression of miRNA can affect the production and function of osteoblasts. Therefore, the abnormal expression of miRNA is closely related to the occurrence of osteoporosis [13], which provides theoretical feasibility for miRNA to become a reliable molecular marker for osteoporosis. However, the current research results of miRNA are still incomplete and sometimes even contradictory. The target genes of some miRNAs that have been proven meaningful and their specific mechanisms of action still need further exploration. In conclusion, further research on the role of miRNA in osteoporosis may help to better understand this disease and potentially discover new molecular markers and therapeutic targets that can assist in the diagnosis and treatment of osteoporosis.

This article aims to summarize the role of miRNA in osteoblasts and discuss the research progress of miRNA as molecular markers for the diagnosis of osteoporosis.

## 2. miRNA in Bone Formation

Osteoblasts are the main players in the process of bone formation. Mesenchymal stem cells are one of the main sources of osteoblasts, and the differentiation of bone marrow mesenchymal stem cells into osteoblasts needs to be strictly regulated by a variety of signaling molecules, such as the Wnt pathway and the BMP pathway [14]. In addition, TGF-β, IGF, Notch, and other signaling pathways also play important roles in the process of osteogenesis. More and more studies have shown that miRNA can directly or indirectly affect the expression of signal molecules in the above-mentioned signaling pathways and participate in the regulation of bone formation.

### 2.1. miRNA in the Wnt Signaling Pathway

**Figure 1:**
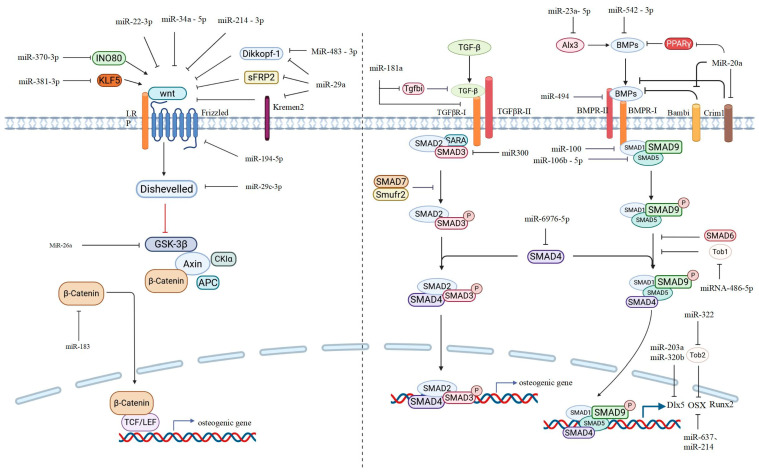
Schematic drawing of Wnt and the TGF-β/BMP pathway implicated in osteoblast differentiation. The miRNAs reported here refer to those that bind to the 3′UTR of the target protein’s mRNA. The solid arrow represent activation or positive regulation, The blocked Arrow means inhibition or negative regulation. The dashed-line blocked arrow indicates inhibition or negative regulation, but the target is not specified.

It is well known that Wnt signaling plays an important role in osteoblast differentiation. Canonical Wnt signaling depends on the enrichment of β-catenin by binding Wnt proteins to transmembrane frizzled receptors and LRP5/6 co-receptors, which recruit Dishevelled proteins in plasma. Dishevelled inhibits β-catenin phosphorylation by GSK3, thereby inhibiting β-catenin ubiquitination and proteasome degradation. As a result, β-catenin is enriched and enters the nucleus to interact with the transcription factors T-cell factor/lymphoid enhancer factor (TCF/LEF) to activate the transcription of target genes [15] (Figure 1). In the non-canonical Wnt signaling pathway, Wnt proteins regulate osteogenesis by binding to FRZ to activate hetero-G-protein and improve intracellular calcium levels. Among the Wnt proteins, Wnt2, Wnt3, Wnt3a, Wnt8, Wnt8b, and Wnt10b function through the canonical Wnt signaling pathway, while Wnt4, Wnt5a, Wnt5b, Wnt6, Wnt7a, and Wnt11 mainly function through the non-canonical Wnt signaling pathway [16]. MiRNAs can negatively regulate osteoblast differentiation and inhibit osteogenesis by targeting the transcription of key genes in the Wnt signaling pathway. For example, Riikka E. Mäkitie et al. demonstrated that miR-22-3p and miR-34a-5p negatively regulate osteogenesis and osteogenic differentiation by targeting Wnt1 mRNA and inhibiting β-catenin expression, thereby inhibiting the formation of calcium nodules during osteoblast differentiation [17]. In the study conducted by Xueling Hu et al., within the rBMSCs miR-214-3p overexpression model, osteogenic-related markers were significantly reduced, and the expression of Wnt3a and β-catenin proteins was also notably downregulated. These findings imply that miR-214-3p may inhibit the osteogenic differentiation of rBMSCs by regulating the Wnt3a/β-catenin signaling pathway [18]. Other studies have shown that osteoclast-derived exosomal miR-214-3p is transferred to osteoblasts to inhibit bone formation. However, Xiaogang Wang et al. suggested that miR-214 inhibits the function of osteoblasts by targeting the 3′-untranslated region (UTR) of ATF4 [19,20]. Wnt5a is involved in the activation of the non-canonical Wnt signaling pathway and works in synergy with the Wnt/β-catenin signal to promote bone formation [21]. MiR-194-5p was shown to suppress Wnt5a expression, thereby inhibiting the activation of the Wnt/β-catenin pathway. However, the authors suggested that miR-194-5p may be involved in the regulation of multiple targets, of which Wnt5a is only one of the main targets affecting MSC differentiation. Therefore, the mechanism of miR-194-5p in MSC differentiation remains to be further explored [22].

MiRNA also plays a significant regulatory role in the downstream signaling molecules of the Wnt signaling pathway. In their study on the physiological functions of CircSmg5, Yue Lu et al. demonstrated that miR-194-5p targets Fzd6 and inhibits the differentiation of BMSC into osteoblasts by suppressing the Wnt signaling pathway [23]. Y.CAO et al. demonstrated that miR-29c-3p can reduce bone loss in rats with DOP via targeted regulation of Disheveled 2 (Dvl2) expression [24]. MiR-26a was shown to target GSK3β and activate Wnt signaling to promote osteogenic differentiation of BMSCs. Interestingly, miR-26a was also shown to inhibit BMP signaling and interfere with osteogenic differentiation of ADSC [25]. The research conducted by Nizhou Jiang et al. suggested that β-catenin has a bidirectional regulatory effect on the stemness of BMSCs. Moreover, miR-183 can directly target and negatively regulate β-catenin, thereby weakening the stemness characteristics of BMSCs [26].

Wnt signaling is negatively regulated by different molecules, such as Dikkopf-1 (Dkk1), Kremen2, and secreted frizzled-related protein 2 (sFRP2), which can interfere with Wnt signaling or the binding of Wnt to its receptors. These negative regulators are direct target genes of miR-29a. miR-29a reduces the expression of these antagonists and enhances Wnt signaling to promote osteogenesis [27]. MiR-483-3p has been demonstrated to promote the proliferation of human osteoblasts and the osteogenic differentiation of pre-osteoblasts, as well as the formation of new bone matrix, by targeting the mRNA of Dikkopf-2 (DKK2) [28]. MiR-106a-5p can target secreted frizzled-related protein 2 (SFRP2), negatively regulate its expression, and inhibit the proliferation and osteogenic differentiation of MC3T3-E1 cells [29].

The INO80 chromatin remodeling complex is crucial in the regulation of transcriptional activation and repression. INO80 can interact with Wdr5 in MSCs and positively regulate classical Wnt signaling transduction [30]. INO80 is the target of miR-370-3p. Experimental results have shown that miR-370-3p inhibits the proliferation and differentiation of osteoblasts by targeting INO80 [31]. KLF5 is an activator of the Wnt/β-catenin signaling pathway and has a promoting effect on the osteogenic differentiation of BMSCs [32]. miR-381-3p has been demonstrated to inhibit osteogenesis during the osteogenic differentiation process of osteoporosis by targeting KLF5 to suppress the Wnt/β-catenin signaling pathway [33].

In addition, some miRNAs have been proven to play significant roles in the process of osteoblast formation and function and are closely related to the Wnt signaling pathway. However, the specific mechanisms of their actions remain unclear. The experiments by Weimin Qiu et al. proved that Wnt3a has an inhibitory effect on the expression of miR-141-3p, and it was found that overexpression of miR-141-3p strongly inhibits the luciferase activity induced by Wnt3a. In addition, miR-141-3p inhibits the ALP activity induced by Wnt3a. Further experiments indicated that miR-141-3p inhibited the proliferation of human mesenchymal stem cells (hMSCs) by targeting the CCND1 gene and arresting cells in the G1 phase of the cell cycle. Nevertheless, current research is unable to account for its inhibitory impact on the Wnt signaling pathway [34]. MiR-107 overexpression activated the Wnt signaling pathway in MC3T3-E1 cells by upregulating the protein levels of Wnt3, β-catenin, and C-My. The target genes directly bound by miR107 were not further studied [35].

### 2.2. miRNA Involved in the TGF-β/BMP Signaling Pathways

There are more than 40 members in the transforming growth factor-β (TGF-β) superfamily, among which the roles of TGF-βs and BMPs in bone formation are widely recognized. This pathway can upregulate the transcriptional expression of RUNX2, DLX5, and OSX, thereby promoting osteoblast maturation [36,37,38] (Figure 1). It is worth noting that TGF-β has a dual effect on osteoblast differentiation. That is, the TGF-β signal promotes the proliferation and early differentiation of osteoblasts, while, at the terminal stage, it inhibits osteogenic differentiation and the expression of osteogenic-related genes [39]. Furthermore, the BMP family includes BMP-2, -6, and -7, which play crucial roles in the differentiation of osteoblasts. BMP-2 can promote the formation of osteocalcin, BMP-7 can facilitate the differentiation of osteoblasts and accelerate calcium mineralization [37], and BMP-6 may have a more stable effect in promoting the expression of osteogenic-related genes [40]. An increasing number of studies have found that miRNAs can target the signaling molecules related to the TGF-β/BMP signaling pathway and participate in the regulation of the proliferation and differentiation processes of osteoblasts. For example, miR-765 can bind to the 3′-untranslated region (UTR) of BMP6 and inhibit its expression, thereby reducing the phosphorylation of Smad1/5/9 and inhibiting the osteogenic differentiation of hMSCs [41]. miR-542-3p exerts a negative regulatory effect on osteogenesis by inhibiting the expression of BMP-7. This, in turn, may lead to the inhibition of the Smad-dependent and non-Smad-dependent BMP-7/PI3K-Survivin signaling pathways. Inhibiting miR-542-3p can accelerate the proliferation and differentiation of osteoblasts. In addition, silencing miR-542-3p leads to increased bone formation in sham operation and ovariectomized (Ovx) mice, as well as improved bone strength and trabecular microstructure [42]. The receptors of TGF-β/BMPs are also one of the targets regulated by miRNAs for bone formation. The research by Tamara Alliston et al. demonstrated that miR-181a can inhibit TGF signaling molecules by targeting the negative regulatory factors Tgfbi and TGF-βR-I/Alk5 (TGF-I type receptor), thereby promoting osteoblast differentiation [43]. The BMP signaling pathway plays a crucial role in the osteogenic differentiation of ADSCs. miR-26a can target Smad1, inhibit the BMP signaling pathway, and negatively regulate the osteogenic differentiation of ADSCs [25]. miR-100 is expressed in mesenchymal progenitor cell lines, and it has also been identified as an endogenous negative regulator of Smad1. It plays a negative regulatory role in the process of differentiation [44]. Overexpression of miR300 in osteoblasts of rat skulls can reduce the protein levels of Smad3, β-catenin, and Runx2. By silencing miR300 in newborn pups and adult rats using Anti-miR300, the inhibitory effect of miR300 on osteoblast differentiation and the expression of the Smad3/β-catenin/Runx2 axis was eliminated. This indicates that miR300 negatively regulates osteoblast differentiation by targeting the crosstalk between Smad3, β-catenin, and Runx2. In animal experiments, the trabecular bone microstructure of the ovariectomized rat model transfected with Anti-miR-300 was improved compared with sham surgery and negative controls, which further confirmed the significant role of miR-300 in the process of steoporosis [45]. MiR-106b-5p and miR-17-5p have been identified as new regulators of Smad5, and they negatively regulate bone formation by targeting Smad5. Furthermore, in sham operation and ovariectomized (OVX) mice, silencing of miR-106b-5p and miR-17-5p increased bone formation and bone mass, thereby improving trabecular bone microstructure [46]. However, another study showed that miR-106a-5p may regulate osteogenesis by inhibiting PTEN and modulating the AKT/NF-κB signaling pathway [47]. Jinming Huang et al. conducted a study exploring the therapeutic effect of pulsed electromagnetic fields (PEMFs) on osteoporosis. They found that PEMF could downregulate miR-6976-5p to reduce bone loss in ovariectomized mice and promote osteogenic differentiation of osteoblast precursor cells treated with hydrogen peroxide. miR-6976-5p targets Smad4. Reducing miR-6976-5p enhances the nuclear transport of phosphorylated Smad1/5/9 by upregulating Smad4, thereby activating the BMP/Smad pathway [48].

Dlx5 is an osteogenic-specific transcription factor whose synthesis is regulated by the BMP signaling pathway. miR-203 and miR-320b negatively regulate osteoblast differentiation induced by BMP-2 by inhibiting Dlx5, thereby suppressing the downstream osteogenic master transcription factors Runx2 and OSX and jointly inhibiting osteoblast differentiation [49]. The experiments conducted by Roland Kocijan et al. also confirmed that Dlx5 is a direct target gene of miR-203a, and they proposed that miR-203a is involved in the delay of osteogenic differentiation, thereby leading to bone loss and the formation of osteoporosis [50].

PPARγ, Bambi, and crim1 are antagonists of the BMP pathway. MiR-20a upregulates the BMP/Runx2 signaling pathway by targeting PPARγ, Bambi, and crim1, thereby promoting osteogenic differentiation [51]. In osteoblasts, the BMP/Smad signaling pathway is negatively regulated by the Tob signal. Exosomes derived from fibroblast-like synoviocytes in rheumatoid arthritis promote osteoblast differentiation by targeting Tob1 [52]. In addition, experiments have shown that Tob2 can bind to the 3-UTR of OSX and regulate its degradation, while miR-322 induces the expression of osteogenic genes by downregulating the expression of Tob2 [53].

Alx3 is a positive regulatory factor for osteoblast differentiation induced by BMP-2. BMP-2 induces an increase in Alx3 gene expression in a time- and dose-dependent manner through the Samd signaling pathway mediated by BMP receptors. Overexpression of Alx3 promotes osteoblast differentiation induced by BMP-2 [54]. Previous experiments have demonstrated that miR-23a-5p negatively regulates the expression of ALX3, thereby influencing the osteogenic differentiation of MC3T3-E cells. However, the specific mechanism by which it regulates osteogenesis still requires further experimental exploration [55].

### 2.3. miRNA Involved in IGF-1 Pathways

**Figure 2:**
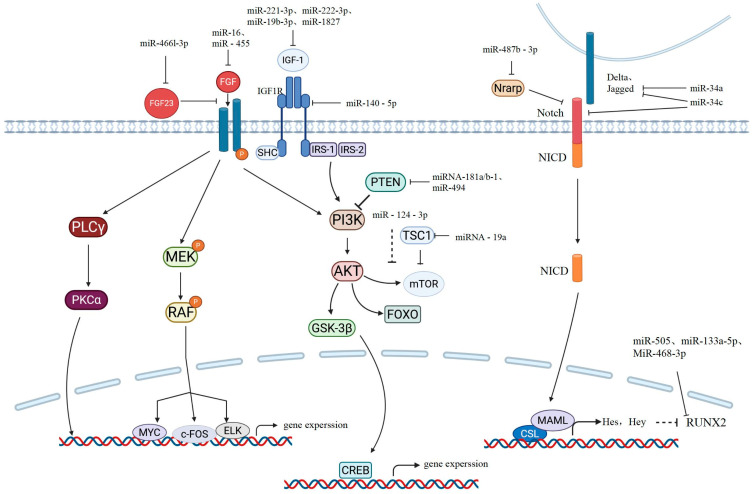
Schematic drawing of the IGF-1, FGF, and Notch pathways implicated in osteoblast differentiation. The miRNAs reported here refer to those that bind to the 3′UTR of the target protein’s mRNA. The solid arrow represent activation or positive regulation, The blocked Arrow means inhibition or negative regulation. The dashed-line blocked arrow indicates inhibition or negative regulation, but the target is not specified.

Insulin-like growth factor 1 (IGF-1) is the most abundant growth factor in the bone matrix. After activation, it mainly transmits signals downward through the PI3K/protein kinase (PK) B (Akt) cascade pathway, regulating the activation of mTORC1, glycogen synthase kinase (GSK)-3, and the FoxO family functions [56] (Figure 2). mTOR is an evolutionarily highly conserved serine–threonine protein kinase that belongs to the PI3K-related kinase family and regulates cell growth and proliferation [57]. It also has an inhibitory effect on the autophagy of cells [58]. Previous studies have demonstrated that IGF-1 activates mTOR through the PI3K-Akt pathway, thereby inducing the differentiation of MSCs into osteoblasts [59,60]. The research conducted by Kang Gan et al. demonstrated that miR-221-3p and miR-222-3p can regulate the activation of ERK by targeting IGF-1, thereby influencing the differentiation of rat mesenchymal stem cells into osteoblasts. Moreover, in a high-sugar environment, silencing miR-221-3p and miR-222-3p can promote the osteogenic differentiation of BMSCs through IGF-1 [61]. The research conducted by ShuangXi Zhu et al. was the first to demonstrate that miR-1827 can regulate osteogenic differentiation. In further experiments, it was proved that miR-1827 directly targets IGF-1 and inhibits the osteogenic differentiation of maxillary sinus mucosal stem cells (MSMSCs) [62]. The research conducted by Yu Yifan et al. revealed that in ovariectomized induced OP rats, the expression levels of miR-19b-3p and IGF-1 were negatively correlated. Their study identified IGF-1 as the target gene of miR-19b-3p and verified the regulation of miR-19b-3p in IGF-1 expression through in vitro and in vivo experiments [63]. Furthermore, experiments have shown that exosomal miR-140-5p can regulate the mTOR pathway by targeting IGF1R, thereby inhibiting the osteogenic differentiation of hMSCs [64]. In a study on the osteogenic differentiation effect of Kaempferol on mesenchymal stem cells, it was found that miR-124-3p can inhibit the PI3K/Akt/mTOR signaling pathway and suppress osteogenic differentiation. Li Gan et al. believed that miR-124-3p targets components of the PI3K/Akt/mTOR signaling pathway (including PIK3CA and AKT2). However, the specific target of miR-124-3p requires further research [65]. miRNA-181a/b-1 has been proven to be able to target the gene PTEN and regulate the Akt pathway, thereby influencing the process of osteogenic differentiation [66]. The tuberous sclerosis complex 1 is an upstream inhibitory protein of mTORC1. In the experiment conducted by Gang Liu et al., it was demonstrated that miRNA-19a, by targeting the 3′-untranslated region of TSC1 mRNA, protects human osteoblasts from the damage caused by dexamethasone [67].

### 2.4. miRNA Involved in Notch Pathways

Notch itself is a transmembrane receptor. When Notch interacts with the membrane-bound ligands Delta or Jagged on the adjacent cell surface, the intracellular domain of Notch is cleaved off from the membrane by γ-secretase and translocated to the nucleus, where it binds to the transcription factor CSL. Then, CSL recruits the co-activator Mastermind-like (MAML) and initiates the transcription of target genes Hes and Hey. The transcriptional activity of Runx2 is negatively regulated by the protein encoded by the Notch target gene Hey1 [15]. In addition, Hey2 transgenic mice showed reduced bone mass and decreased alkaline phosphatase expression in vitro. Hes1 determines bone mass and bone structure. The inactivation of Hes1 can increase the volume of trabecular bone in vivo and enhance the expression of osteoblast-related genes [68]. MiR34c and miR-34a play significant roles in the process of osteoblast formation. miR-34c directly targets multiple components of the Notch signaling pathway, including Notch1, Notch2, and Jag1, while miR-34a exerts its effect by targeting Jag1 [69,70]. In the study by Li-Jue Ren et al., when OVX rats overexpressed miR-210, the protein levels of VEGF, Notch1, and Jagged1 significantly increased. However, when miR-210 was inhibited, the above protein levels significantly decreased. This suggests that overexpression of miR-210 may improve osteoporosis in menopausal rats by activating the VEGF/Notch1 signaling pathway. However, the target genes of miR-210 have not yet been explored [71]. In addition, Nrarp is an inhibitor of the Notch signal. It can suppress the Notch-1 signal by stabilizing LEF-1. Experiments have verified that Nrarp is a direct target gene of miR-487b-3p. miR-487b-3p negatively regulates osteogenesis by inhibiting the expression of Nrarp, thereby suppressing the Runx-2 and Wnt signals [72].

### 2.5. miRNA Involved in FGF Pathways

Fibroblast growth factors (FGFs) have been proven to be a crucial signaling pathway in the process of bone formation. Briefly, after the FGF signal binds to the fibroblast growth factor receptor (FGFR), FGFR undergoes phosphorylation, activating the PLCγ/PKCα, MEK/ERK, and PI3K/AKT signaling pathways and thereby promoting the expression of osteoblast-related genes and cell proliferation [73,74]. In recent years, many miRNAs have been discovered to be able to target FGF signaling and its downstream signaling molecules to exert effects on the bone formation process. For example, in a study on the mechanism by which magnesium promotes osteogenic differentiation of mesenchymal stem cells, it was found that miR-16 targets FGF2 and regulates the ERK/MAPK pathway mediated by FGF2 to inhibit the osteogenic process [75]. The research conducted by Lingzi Niu et al. revealed that mice lacking miR-455 exhibited an increase in the length of long bones and vertebrae, an increase in trabecular bone, and a decrease in porosity, and further experiments confirmed that FGF18 was a direct target of miR-455. miR-455 may exert its inhibitory effect on bone formation by regulating FGF18 [76]. FGF23 is one of the members of the FGF family. It has been proven to have inhibitory effects on the osteogenic process of BMSCs and the mineralization of mature osteoblasts. The research by Xiang Zhang et al. suggested that miR-466l-3p, targeting FGF23, promotes the osteogenic differentiation of hBMSCs through the PI3K/AKT/mTOR pathway. The miR-466l3p/FGF23 axis may be a potential biomarker for osteoporosis [77].

### 2.6. miRNA Involved in Transcription Factors of Osteoblastogenesis

In the process of bone formation, transcription factors such as Runx2 and Osx are also important for achieving osteogenic regulation. Currently, many miRNAs have been discovered to affect the expression and activity of Runx2. The experiments by Weihua Li et al. identified the target gene of miR-505 as RUNX2, and their further cell experiments verified that the expression of miR-505 was downregulated during the osteogenic differentiation process of MC3T3-E1 cells. Transfection of miR-505 could inhibit the expression of osteogenesis-related genes in MC3T3-E1 cells. When miR-505 is inhibited, the expression of osteogenic marker genes is upregulated [78]. Wei Zhang et al. found that miR-133a-5p mimics significantly reduced the expression of collagen I, OCN, and OPN in MC3T3-E1 cells. An miR-133a-5p inhibitor significantly promoted the expression of collagen I, OCN, and OPN in MC3T3-E1 cells. In addition, the luciferase reporter assay confirmed the targeted binding relationship between miR-133a-5p and RUNX2 [79]. MiR-468-3p has been proven to be a novel Runx2 regulatory factor, exerting a negative regulatory effect on the osteogenic process [80]. The specific transcription factor of osteocytes, Osterix, has been confirmed to be a direct target of miR-637. This miRNA significantly enhances the differentiation of hMSCs into adipocytes by directly inhibiting the expression of Osx while inhibiting osteogenic differentiation [81]. MiR-214 has been identified as an Osx regulatory factor, which inhibits the important role played by C2C12 bone differentiation [82].

## 3. miRNAs in Osteoporosis Diagnosis

An appropriate biomarker for osteoporosis should possess characteristics such as simple sample collection, non-invasive measurement, specificity for bone metabolism, correct correlation with standard techniques for bone remodeling analysis, and the ability to respond to the treatment of diseases affecting bone metabolism. Currently, a number of microRNAs, particularly circulating miRNAs, have been demonstrated to possess the ability to distinguish osteoporosis patients and to identify individuals at high risk of fracture in osteoporosis. Moreover, some miRNAs have been identified as biomarkers for osteoporosis treatment [83]. The following section will discuss the potential value of miRNAs as molecular markers for osteoporosis diagnosis (Table 1).

### 3.1. miRNA Plays a Significant Role in the Diagnosis of Osteoporosis and the Prediction of the Risk of Brittle Fractures

The research by Zhang et al. suggested that miR-23a-5p is upregulated in the serum of patients with PMOP. The AUC value for differentiating OP patients from non-OP individuals was 0.939, with a 95% CL of 0.905–0.974. It had a high sensitivity (80.8%) and specificity (91.7%). Although the sample size of their experiment was limited and further large-scale clinical validation is needed, miR-23a-5p is still considered a promising biomarker [55]. Yang et al. demonstrated that miR-370-3p plays a significant role in regulating the proliferation, apoptosis, and differentiation of osteoblasts. They also discovered that this miRNA is upregulated in patients with osteoporosis. The ROC curve analysis yielded an AUC of 0.884, a sensitivity of 79.69%, and a specificity of 87.69%. The authors proposed that the early identification of the disease could be achieved by detecting the expression levels of miRNA in blood, saliva, or other body fluids. However, the source of miRNA in serum and the inducing factors for its upregulation remain unclear. The next step of the research will involve expanding the sample size and conducting independent verification by combining multi-center data to explore the source of miR-370-3p in serum and further verifying the relationship between this miRNA, estrogen, vitamin D3, and osteoporosis [31]. In the study by Zhang et al., it was found that in a diabetic osteoporosis model induced by high-glucose stimulation of rat cranial osteoblasts (ROBs), miRNAs such as miR-330-5p, miR-185-5p, and miR-190a-3p showed differential expression. However, the most significantly differentially expressed was miR-702-5p. They proposed that the miRNA-702-5p/OGN/Runx2 signaling axis might play a role in DOP and that it could not only serve as a diagnostic marker for DOP but also be a therapeutic target for other forms of osteoporosis [58]; however, further research is needed to verify the effects of miR-702-5p in the body [84]. In addition, some experiments have shown that compared with healthy volunteers, the level of miR-33a-3p in the serum of patients with osteoporosis is significantly higher. miR-33a-3p negatively regulates the level of IGF2 in hBMSCs and affects the differentiation of osteoblasts. These findings suggest that miR-33a-3p can serve as a plasma biomarker and therapeutic target for postmenopausal osteoporosis [85]. Zheng et al. conducted a WGCNA analysis and found that the expression level of circulating miR-107 was significantly lower in osteoporosis patients compared to a healthy control group. Through ROC analysis, miR-107 provided an AUC > 85% for differentiating female osteoporosis patients from healthy controls, as well as an AUC > 85% for differentiating female osteoporotic patients with vertebral compression fractures from those without vertebral compression fractures. However, the target genes of miR-107 have not yet been explored, and this is their next experimental direction [35]. Lin et al. verified that miR-1303 was expressed at a lower level in patients with osteoporosis and proposed that the KCNMA1-AS1/miR-1303/COCH axis is a promising biomarker and therapeutic target for the diagnosis and treatment of osteoporosis. Even though the sample size of their study was small, it is the first report on the research of miR-1303 in osteoporosis [86]. Ajda Bedene’s research analyzed miRNA isolated from the plasma samples of 74 postmenopausal women. It was determined that the expression of miR-148a -3p was significantly elevated in the group of patients with osteoporosis and affirmed its potential value as a biomarker for identifying pathological changes related to osteoporosis [87]. Samantha Lincoln’s research indicated that miR-148a-3p is a mediator factor for osteoporosis following spinal cord injury and a potential future therapeutic target. However, the sample size of her study was relatively small, and due to the design of the study, it was not possible to verify the level of miR-148-3p in independent samples [88]. The expression of miR300 was significantly elevated in patients with osteoporosis, with an AUC of 0.9689, a 95% CI ranging from 0.9275 to 1.010, and *p* < 0.05. The study suggests that using miR300 as a low-cost, effective, and less invasive biomarker for detecting osteoporosis may be a reasonable alternative to dual-energy X-ray absorptiometry as a detection method [45]. In Suzan Magdy Ismail’s research, miRNA-208a-3p, miRNA-155-5p and miRNA-637 were also identified as miRNAs with diagnostic significance for osteoporosis. Interestingly, in the study, the experiment also included premenopausal osteoporosis. The experiment found that the serum miRNA-208a-3p of premenopausal patients was significantly upregulated, while miRNA-155-5p was significantly downregulated. The level of miRNA-637 in premenopausal patients showed a non-significant decrease compared to the respective control group and premenopausal osteoporosis patients. The three miRNAs studied were significantly upregulated in postmenopausal patients [89]. MiR-208a-3p has also been reported to be associated with menopause, showing a negative correlation with E2, a positive correlation with FSH and LH, and a significant upregulation in postmenopausal women with osteoporosis. It has significant diagnostic potential. However, the study suggested that miR-208a-3p exerts its effect on bone metabolism by mediating osteoclast activation [90]. In Clara Pertusa’s study, the serum level of miR-155-5p was proven to be able to distinguish a group of patients with hip fragility fractures from a healthy control group [91]. This result further confirms the significant role of miRNA in the diagnosis of osteoporosis.

### 3.2. miRNA with Other Diagnostic Techniques for Osteoporosis

The expression of miRNA is highly correlated with the current diagnostic analysis techniques for osteoporosis. For instance, miR-210 shows a significant decrease in expression in the femoral tissue of OVX rats. Overexpression of miR-210 can significantly increase BMD, BMC, BV/TV, and Tb.Th, while significantly reducing BS/BV and Tb.Sp. miR-210 has been proven to affect the levels of bone turnover markers, such as BALP, CTX-1, PINP, and OCN in the serum of OVX rats, as well as the expression of osteogenic-related markers (Runx2, OPN, and COL1A1). However, its target genes and the specific mechanisms by which it affects osteogenesis still require further experimental exploration [71]. In addition, the research conducted by Ursula Heilmeier et al. demonstrated that in postmenopausal women with T2D, the low expression of miR-19b-1-5p was significantly associated with a reduced risk of fragility fractures. The low expression of miR-203a and miR-31-5p was also significantly correlated with the occurrence of fragility fractures. Moreover, it was proposed that the AUC for diagnosing fragility fractures using these three miRNAs was 0.722. The clinical parameters, such as aBMD, Clinical FRAX, or FRAX aBMD, in identifying new fractures had comparable diagnostic accuracy. However, since the postmenopausal women with T2D selected for the study were all over 76 years old, the results of the study may not be applicable to younger postmenopausal women with T2D or T2D men in the research on fragility fractures [92]. In another study reporting on the circulating miRNAs of patients with impaired WNT signaling, the differential expression of miR-31-5p was also identified, and it was speculated that this miRNA might have potential application value in the diagnosis and treatment of osteoporosis [17]. Another report indicated that miR-203a-3p and miR-181c-5p may have potential value as biomarkers for PLS3-induced osteoporosis [93]. miR-29cb2 has been proven to be selectively and effectively transferred to the peripheral blood during osteoporosis. The relative expression level of miR-29cb2 in the peripheral blood of osteoporosis patients is significantly higher than that of patients with reduced bone mass. The AUC of miR-29b2 is 0.733 (95% CI: 0.496–0.970), and the AUC of miR-29c is 0.781 (95% CL: 0.571–0.990). This experiment also compared the diagnostic efficacy of four clinical markers for osteoporosis, namely, osteocalcin (AUC: 0.683), parathyroid hormone (AUC: 0.515), collagen I degradation product (AUC: 0.595), and 25-OH-VitD (AUC: 0.556). The results showed that miR-29c has higher sensitivity and specificity. However, the authors believed that more disease models, longer observation times, and more patients are needed to verify the role of miR-29cb2 in bone mass reduction [94]. The research by Lu et al. indicated that the serum miR-206 level was positively correlated with BMD, and they believed that miR-206 had the ability to distinguish postmenopausal female patients with osteoporosis from those without it. The ROC curve results showed an AUC of 0.860, with a sensitivity of 73.0% and a specificity of 87.7% [95].

### 3.3. The Combined Diagnostic Model of miRNA

The combined diagnostic model of miRNAs may enhance their diagnostic efficacy. The research by Ursula Heilmeier et al. also proposed that the combination of miR-19b-1-5p, miR-203a, and miR-31-5p with aBMD (AUC: 0.75695%, CI: 0.680, 0.823) would have a better diagnostic efficacy than using aBMD alone (AUC: 0.666, 0.585, 0.741) [92]. The research conducted by Senay Balci et al. identified that the expression levels of miR-21-5p, miR-34a-5p, miR-210, miR-122-5p, miR-125b-5p, miR-133a, miR-143-3p, miR-146a, miR-155-5p, and miR-223 were all decreased in the serum of osteoporosis patients. In the receiver operating characteristic curve analysis of the subjects in their study, the single and combined effects of miRNA in diagnosing osteoporosis were determined. Among them, the combination of miR-34, miR-125, miR-133, and miR-210 (AUC: 0.882) demonstrated superior diagnostic efficacy compared to other combinations [96].

### 3.4. The Response of miRNA to the Therapeutic Effect of Osteoporosis Treatment

Furthermore, miRNA also undergoes corresponding changes after OP treatment. For instance, in the serum of osteoporosis patients treated with triptolide for 3 months, miR-33a-3p significantly decreased [85]. The levels of miR-181c-5p and miR-497-5p in the serum of postmenopausal women with reduced bone mass or osteoporosis decreased, but they increased in the subjects treated with bisphosphonates combined with calcitriol. Moreover, miR-181c-5p and miR-497-5p were significantly downregulated in the bone tissues of aging and OVX mouse models. The study was also verified at the cellular level. The authors proposed that circulating miR-181c-5p and miR-497-5p may serve as potential biomarkers for monitoring the efficacy of anti-osteoporosis treatment or for diagnosis [97]. The expression level of miR-203a-3p in the tissues and serum of OVX animals reversed after treatment with teriparatide and zoledronic acid. This result indicates that peripheral blood miRNAs can respond to bone loss and treatment. Additionally, the author believed that whether estrogen replacement can rescue or reverse the effects of estrogen deficiency on miRNAs is an important issue, which will be explored in further studies [50].

## 4. Conclusions

In conclusion, miRNAs, as important molecules for post-transcriptional regulation of genes, possess high conservation, stable detection, and accessibility, and thus have broad prospects in research as disease prediction markers. Increasing evidence indicates that miRNAs are involved in various processes of bone metabolism and osteoporosis onset and can reflect the bone metabolism status from multiple aspects. Therefore, miRNAs have significant value as molecular markers for osteoporosis diagnosis. However, there are still many miRNAs whose target genes and specific mechanisms involved in osteoporosis have not been explored, an there is a lack of sufficient clinical verification to prove whether these miRNAs can be used as biomarkers in clinical applications. In the future, it will still be necessary to further improve the miRNA expression profile of osteoporosis, explore its role in the pathogenesis of osteoporosis, and complete clinical verification, so as to apply it to clinical diagnosis, prognosis judgment, and treatment. Moreover, combining the identified miRNAs with traditional diagnostic methods to form new diagnostic models may be able to improve diagnostic efficiency.

## Figures and Tables

**Table 1:** Summary of miRNAs, their target genes, expression profile, and potential for diagnosing osteoporosis or the risk of fracture.

miRNA	Target Gene	Sample Source	Sample	Expression in OP	AUC	Sensitivity	Specificity	95%CL	Reference
miR-19b-1-5p	-	T2D postmenopausal women	serum	downregulated	-	-	-	-	[1]
miR-23a-5p	ALX	postmenopausal women	serum	Upregulated	0.939	80.8%	91.7%	0.905~0.974	[2]
miR-29c	HIF-3a	OVX mice	peripheral blood	Upregulated	0.781	-	-	0.571~0.990	[3]
miR-29b2	HIF-3a	OVX mice	peripheral blood	Upregulated	0.733	-	-	0.496~0.970	[3]
miR-31-5p	-	T2D postmenopausal women	serum	Upregulated	-	-	-	-	[1]
miR-107	-	postmenopausal women	serum	downregulated	0.866	-	-	0.8082~0. 9644	[4]
miR-33a-3p	IGF2	postmenopausal women	serum	Upregulated	-	-	-	-	[5]
miR-148a-3p	WNT1 WNT10B KDM6B DNMT1 IGF1	spinal cord injury patients	serum	Upregulated	-	-	-	-	[6]
miRNA-155-5p	SOCS1	premenopausal women postmenopausal women	serum	Downregulated Upregulated	0.900 0.828	94.29% 80%	77.14% 80%	- -	[7]
miR-181c-5p	TGF-β Tgfbi TβR-I	postmenopausal women	serum	downregulated	0.87	88%	71%	0.75~1.0	[8]
miR-203a	-	T2D postmenopausal women	serum	Upregulated	-	-	-	-	[1]
miR-206	HDAC4	postmenopausal women	serum	downregulated	0.860	73.0%	87.7%	-	[9]
miRNA-208a-3p	ACVR1	premenopausal women postmenopausal women	serum	Upregulated Upregulated	0.816 0.851	77.14% 80%	82.86% 82.86%	- -	[7]
miR-210	-	OVX rats	femoral tissues	downregulated	-	-	-	-	[10]
miR300	Smad3	osteoporotic patients	serum	Upregulated	0.9689	-	-	0.9275~1.010	[11]
miR-370-3p	INO80	postmenopausal women	serum	Upregulated	0.884	79.69%	87.69%	-	[12]
miR-483-3p	dikkopf 2	osteoporotic patients	bone tissue	downregulated	-	-	-	-	[13]
miR-497-5p	IL-1	postmenopausal women	serum	downregulated	0.92	94%	79%	0.83-1.01	[8]
miRNA–637	Osx	premenopausal women postmenopausal women	serum	downregulated Upregulated	- 0.814	- 77.14%	- 85.71%	-	[7]
miR-702-5p	OGN	rats	ROBs	Upregulated	-	-	-	-	[14]
miR-1303	COCH	postmenopausal women	serum	downregulated	-	-	-	-	[15]

## Data Availability

No new data were created or analysed in this study. Data sharing is not applicable.

## Abbreviations

The following abbreviations are used in this manuscript:

miRNAs	MicroRNAs
3′-UTR	3′-untranslated region
OP	Osteoporosis
BTMs	Bone turnover markers
BMD	Bone mineral density
DXA	Dual-energy X-ray absorptiometry
FRZ	Transmembrane frizzled receptor
rBMSCs	Rat mesenchymal stem cells
ADSC	Adipose tissue-derived mesenchymal stem cell
hMSCs	Human mesenchymal stem cells
MSMSCs	Mouse mesenchymal stem cells
AUC	Area under the curve
ROC	Receiver operating characteristic curve
DOP	Diabetic osteoporosis
OVX	Ovariectomy
Tb.Sp	Trabecular separation
BV/TV	Bone volume/total volume
Tb.Th	Trabecular thickness
BS/BV	Bone surface area/bone Volume
SOCS1	Suppressor of cytokine signaling 1
KDM6B	Lysine demethylase 6B
DNMT1	DNA methyltransferase 1
ALX	Receptor tyrosine kinase
HDAC4	Histone deacetylase 4
COCH	Cochlin
